# Co-inhibition of Notch1 and ChK1 triggers genomic instability and melanoma cell death increasing the lifespan of mice bearing melanoma brain metastasis

**DOI:** 10.1186/s13046-025-03411-w

**Published:** 2025-05-28

**Authors:** Varsha Thakur, Vijay S. Thakur, Dazhi Wang, Juliano Tiburcio de Freitas, Anna Bianchi, Luis Alberto Nivelo, Oliver Umland, Scott M. Welford, Barbara Bedogni

**Affiliations:** 1 Dr. Phillip Frost Department of Dermatology and Cutaneous Surgery, University of Miami Miller School of Medicine, Sylvester Comprehensive Cancer Center, Miami, FL 33136 USA; 2 Department of Radiation Oncology, University of Miami Miller School of Medicine, Sylvester Comprehensive Cancer Center, Miami, FL 33136 USA; 3 Division of Surgical Oncology, Dewitt Daughtry Department of Surgery, University of Miami Miller School of Medicine, Miami, FL 33136 USA; 4 Diabetes Research Institute, University of Miami Miller School of Medicine, Miami, FL 33136 USA

**Keywords:** Melanoma brain metastases, Anti-Notch1 antibody, ChK1 inhibition, Genomic instability, DNA damage

## Abstract

**Background:**

Melanoma brain metastases (MBM) are a leading cause of death in patients with advanced disease. MBM treatment relay on targeted and immunotherapy and on stereotactic radiosurgery as gold standard. Life expectancy has improved significantly with these therapies however, targeted therapy is short lived and only about half of the patients respond to immunotherapy, while radiation is limited by melanoma cells intrinsic resistance to DNA damage. New therapeutic approaches are therefore needed to treat MBM. Here we investigate a new role of Notch1 in genomic instability and demonstrate that blockade of both Notch1 and the DNA repair factor ChK1 causes extensive DNA damage and tumor cell death increasing survival in MBM bearing mice.

**Methods:**

Anti-Notch1 (anti-N1) was previously described. Prexaserib, a ChK1 inhibitor, is currently in clinical trials. K457 and A375 melanoma cells were used. RNA sequencing was performed in K457 cells treated with anti-N1 and Gene Set Enrichment Analysis performed. DNA damage was evaluated by a DNA fiber assay to assess replication fork speed; and γH2AX foci count and neutral comet assay to quantify double strand breaks. Cell survival was evaluated by trypan blue and a colony formation assay. Luciferase expressing A375 cells were orthotopically inoculated in the right cerebral cortex of athymic nude mice, for in vivo evaluation of a therapy with anti-N1 and prexasertib. Survival was assessed by Kaplan-Meyer survival curves and significance assessed by a Log-rank test.

**Results:**

Notch1 blockade caused genomic instability by reducing histone availability, leading to DNA replication stress and DNA damage. This in turn, resulted in the activation of the DNA Damage Response pathway ATR/ChK1 to counter the damage. Co-inhibition of Notch1, via anti-N1, and ChK1, via prexasertib (prex), exacerbated DNA damage increasing melanoma cell death. Importantly, combination anti-N1/prex significantly improved survival of mice bearing MBMs.

**Conclusions:**

A therapy with anti-N1/prexasertib could represent a novel treatment strategy, alone or in combination with current treatment regimens, for melanoma brain metastases.

**Supplementary Information:**

The online version contains supplementary material available at 10.1186/s13046-025-03411-w.

## Introduction

Melanoma is the third most common source of intracranial metastases in adults after lung and breast cancer. patients with stage IV melanoma have a 20–30% incidence of brain metastases (BM) in year 1 from diagnosis, 30–40% incidence at 3 years and up to 73% incidence at autopsy. Survival for patients with untreated, symptomatic melanoma brain metastases (MBM), ranges from several weeks to a few months; and the prognosis for MBMs is worse than in patients with BMs from other solid tumors [1].

The American Society for Radiation Oncology (ASTRO) generally recommends stereotactic radiosurgery (SRS) for 1–4 brain metastases especially if symptomatic. Asymptomatic BM may be first treated with eligible targeted and immunotherapy prior to SRS. Whole brain radio-therapy (WBRT) is recommended in patients ineligible for surgery or SRS but who have favorable prognosis [2]. In targeted therapy, it has been shown that 58% of MBM patients respond to BRAFi/MEKi combinations but the treatment is short lived [3, 4]. Immunotherapy with single-agent ipilimumab induces response in approximately 20% of patients, while the combination of anti-CTLA-4 + anti-PD1 induces durable responses in 55% of patients [5, 6]. Studies have also shown an increased overall survival (OS) in patients receiving SRS and immunotherapy versus SRS alone [5]. However, immunotherapy when combined with SRS, increases radiation necrosis, leading to the worsening of cognitive impairments [5]. Also, recurrence is still high after these treatments due to melanoma intrinsic resistance to radiation; and only about half of the patients with MBM respond to immunotherapy. Therefore, new therapeutic approaches are still needed to improve response and overall survival of patients with MBMs.

Notch1 is an evolutionarily conserved signaling cascade with critical roles in the maintenance of melanocyte stem and precursor cells homeostasis [7]. Notch1 is involved in virtually all hallmarks of melanoma. It is over-expressed in over 60% of melanomas; it is required for growth and survival of melanoma cells [8–13]; and it promotes a tolerogenic tumor microenvironment which reduces the efficacy of immuno-checkpoint inhibitors (ICI) [14]. Indeed, we recently demonstrated that selective targeting of Notch1 via a novel neutralizing monoclonal antibody (anti-N1)**,** significantly enchased the efficacy of anti-PD1 in melanoma syngeneic models [15].

Here we show, for the first time, that selective blockade of Notch1 causes genomic instability by reducing histone availability, which leads to DNA replication stress and subsequent DNA damage. This in turn, leads to the activation of the DNA Damage Response (DDR) pathway ATR/ChK1 to counter the damage. Concomitant blockade of both Notch1, via anti-N1, and ChK1, via prexasertib (prex), a ChK1/2 inhibitor currently in several phase 2 clinical trials [16–18], exacerbates DNA damage increasing melanoma cell death. Importantly, combination anti-N1/prex significantly improves survival of mice bearing MBMs. This new combination therapy could represent a novel treatment strategy, alone or in combination with current treatment regimens, for melanoma brain metastases.

## Materials and methods

### Cell lines and reagents

Human K457 and A375 melanoma cells and normal human melanocytes were a gift from Dr Marianne Broome Powell, (Stanford University) [13, 19]. BJ fibroblasts were purchased from ATCC (Manassas, Virginia). Melanoma cells and fibroblasts were maintained in DMEM (Dulbecco's modified Eagle's medium) supplemented with 10% fetal calf serum, 1% glutamine and 1% penicillin–streptomycin (Pen/Strep). Human melanocytes were grown in poly-L-lysine-coated plates using MelM melanocyte medium (ScienCell Research Laboratories). Anti-N1 was produced in house as described in [15] and used at 50–100 µg/ml in culture. Prexasertib HCl (LY2606368), was purchased from Selleck Chemicals and used at 5 nM in culture.

### Immunoblot analysis

Cells (1 × 10^6^) were plated in complete media containing vehicle (DMSO) or anti-N1 (100 µg/ml), or prex (5 nM), and collected at 48 or 72 h after treatment. Total protein was extracted with urea lysis buffer (9 M urea, 75 μM Tris–HCl, pH 7.5, and 100 μM 2-mercaptoethanol). 40–50 μg protein per sample was separated by 8–10% SDS-PAGE and transferred onto PVDF membranes. Antibodies used were: anti cleaved-Notch1 (Novus biologicals, NB100-78486), Histone H2 A.X (clone D17 A3 XP, Cell signaling Technology, cat #7631); Histone H3 (clone D1H2, Cell signaling Technology, Cat #4499); Histone H2B, (Proteintech cat #15,857–1-AP); Histone H4 (Proteintech cat # 16,047–1-AP)**,** anti-phospho-ChK1^S345^ (clone 133D3, Cell signaling Technology, cat #2348), anti-phospho-ChK1^S296^ (clone D3O9 F, Cell signaling Technology, cat #90,178) and total ChK1 (clone 2G1D5, Cell signaling Technology, cat #2360). Loading was normalized with anti–β-actin (cat #A2228) or α-tubulin (cat #T6199), both from Sigma-Aldrich, Inc, St. Louis, MO, US.

### Cell survival – Trypan Blue assay

5 × 10^4^ A375 and K457 cells were seeded in 24-well plates in complete media, in triplicate, and treated with aN1 (100ug/ml) and prex (5 nM) alone or in combination. Cell survival was evaluated by trypan blue exclusion, by counting alive and dead cells with the TC 20 automated cell counter (Biorad) after 48 h.

### Colony formation assay

A total of 500 A375 cells per well, in triplicate, were seeded in a 12 well plate in complete media containing anti-N1 (50 µg/ml), prex (5 nM) or both. After 10–12 days incubation, colonies were stained with crystal violet and scored using GelCount (Oxford Optronix) as previously described [15].

### DNA fiber assay

DNA fiber assay to determine replication fork (RF) speed, was performed as previously described [20]. Cells were pulse-labeled with 250 μmol/l CldU for 30 min followed by a second pulse with 50 μmol/l IdU (Sigma) for another 30 min. Cells were then lysed [0.5% SDS, 200 mmol/l Tris–HCl (pH 7.4), 50 mmol/l EDTA] and dropped and spread onto an uncoated glass slide and let dry. DNA spreads were fixed with a 3:1 solution of methanol-acetic acid for 10 min, let dry and then placed in 70% ethanol at 4 °C for 1 h. DNA was denatured with 2.5 mol/l HCl for 30 min at 37 °C. Slides were blocked in 1% BSA and then incubated with mouse anti-BrdU antibody (BD Biosciences) and rat anti-CldU antibody (Abcam). Alexa Fluor 594, or Alexa Fluor 488 (Thermo Fisher Scientific) secondary antibodies were used. DNA fibers were viewed at × 100 magnification on a Keyence BZ-X800 microscope. Signals were measured using ImageJ as previously described [20, 21].

### Comet assay

Comet assays were performed as previously described [20, 21]. Briefly, 1 × 10^6^ cells were seeded in 60 mm cell culture dishes in complete media containing DMSO, anti-N1 (100 µg/ml), prex (5 nM) or a combination of anti-N1/prex, for 72 h. Cells were trypsinized to obtain single cell suspensions, counted and the number of cells was normalized among samples. 5 μl of each cell suspension was mixed with pre-melted low melting agarose and plated on glass slides provided in the kit. After solidification at 4 °C, slides were immersed in cold lysis buffer. Electrophoresis was carried out at 21 V for 45 min using neutral electrophoresis buffer (1 × TBE). Slides were washed and then fixed in ethanol (70%) followed by drying at 37 °C overnight. Slides were then stained with Sybr green DNA gel stain (Invitrogen, cat# S33102, 1:10,000). Comets were imaged at × 10 magnification. Comet analysis was done using Comet Score (TriTek). At least 200 comets were included per condition.

### Immunofluorescence

Cells were fixed in 3% formaldehyde after 72 h treatment and stained using standard procedures with γH2 AX antibody (anti-phospho-histone H2 A.X (Ser139) clone JBW301, Millipore, cat# 05–636, 1:500) and secondary Alexa-Fluor 594 anti-mouse (Invitrogen, cat# A11032, 1:500). For mitotic catastrophe, fixed cells were stained using anti-Ki67 (clone D3B5, cat #9129, at 1:200) and cleaved-caspase 3 (Asp175, cat #9661, at 1:200), both from Cell Signaling Technology. Slides were mounted in Vectashield with DAPI (Vector Laboratories cat #H-1200–10). Immunofluorescence was observed at × 400.

### Cell cycle assay

10^6^ cells were seeded in 60 mm dishes containing anti-N1 (100 µg/ml), prex (5 nM) or both, and incubated for 72 h. At the last hour of incubation, 10 mM EdU (ethynyl-2 deoxyuridine) was added to each plate. Click-iT™ EdU Alexa Fluor™ 647 Flow Cytometry Assay Kit (C10424) was used to detect cells in the s phase as per manufacturer’s instructions.

### RNA-sequencing and data analysis

Total RNA from 10^6^ K457 cells treated for 48 h with anti-N1 (50 µg/ml), was extracted using the RNeasy Mini Kit (Qiagen, Cat# 74104) according to the manufacturer’s instructions. Before library construction, all samples were assessed for RNA purity via 260/280 ratio using Nanodrop, then RNA integrity and potential DNA contamination using agarose gel electrophoresis, and RNA integrity again using the Agilent Bioanalyzer 2100. mRNA was purified from total RNA using poly-T-oligo-attached magnetic beads and the mRNA were fragmented randomly by addition of fragmentation buffer. For NEB library preparation using NEBNext® Ultra™ RNA Library Prep Kit for Illumina®, briefly, the first strand cDNA was synthesized using random hexamer primer and M-MuLV Reverse Transcriptase (RNase H-) and second strand cDNA synthesis was subsequently performed using DNA polymerase I and RNase H. The double-stranded cDNA was then purified using AMPure XP beads and the remaining overhangs of the purified double-stranded cDNA were converted into blunt ends via exonuclease/polymerase activities. After adenylation of 3’ ends of DNA fragments, NEBNext Adaptor with hairpin loop structure was ligated to prepare for hybridization. In order to select cDNA fragments of preferentially 150 ~ 200 bp in length, the library fragments were purified with the AMPure XP system (Beckman Coulter, Beverly, USA). Finally, the final library was obtained by PCR amplification and purification of PCR products by AMPure XP beads. The libraries were pooled in equimolar amounts and sequenced in paired end 150 bp reactions on the Illumina NovaSeq 6000.

The raw FASTQ data were processed through an in-house bioinformatics pipeline including quality and adapter trimming with TrimGalore version 0.6.1 (https://www.bioinformatics.babraham.ac.uk/projects/trim_galore/), alignment to the human genome hg38/GRCh38 and gene quantification was done with the STAR algorithm v2.5.2 [22]. The differential expression analysis and GSEA (gene set enrichment analysis) were performed using iDEP2.0 and ShinyGO [23].

### Bulk transcriptomic analysis

Normalize count tables and expression matrices were downloaded for the GSE200217, and GSE50496 datasets. Differential gene expression analysis was performed using the limma R package (v3.58.1). A design matrix was constructed to model the experimental groups. Linear models were fitted using lmFit, followed by empirical Bayes moderation with eBayes. Differentially expressed genes (DEGs) were identified based on an adjusted *p*-value < 0.05 (Benjamini–Hochberg correction). Pathway enrichment analysis was performed via fGSEA (fast Gene Set Enrichment Analysis, v1.28) to the ranked genes based on their foldchange derived from the limma analysis. The ranked gene list was used to test for enrichment against the MSigDB (Molecular Signatures Database) Hallmark database. Gene set significance was determined using adjusted *p*-values (< 0.05) and normalized enrichment scores (NES). The results were visualized using bubble plots obtained with ggplot2 R package v3.5.1.

### Single-nuclei (sn) RNAseq analysis

snRNAseq files (*n* = 25) from GSE200218 dataset were combined in a Seurat object (Seurat R package, v5.1). Samples were then stratified based on biopsy site (brain or peripheral). Notch1 signaling signature was firstly evaluated by creating a module score (AddModuleScore function, Seurat) with a gene set obtained from the “Hallmark_Notch_Signaling” (Hallmark database, MSigDB). Differential gene expression between Brain vs Peripheral sites within the Tumor Cell cluster was performed (FindMarkers function, Seurat), followed by pathway enrichment analysis via fGSEA as described in the method section: Bulk transcriptomic analysis.

### In vivo studies

Animal studies were performed in accordance with University of Miami institutional guidelines. Eight-week-old female athymic nude mice (Charles River) were stereotactically injected intracranially with luciferase-expressing A375 cells (10^5^), into the right cerebral cortex at a depth of 3 mm. Tumor growth was monitored twice weekly and quantified using bioluminescent imaging (BLI). Signal intensity was measured as photon counts within a Region Of Interest (ROI). One day post inoculation, mice were equally distributed into groups of treatment so that each group contained mice with similar tumor burden, measured by BLI. Mice than received anti-N1 (100 mg/Kg) daily and prex (10 mg/Kg) for three days followed by four days holiday period [24]. Animal appearance, behavior, and weight were monitored to evaluate tumor progression as per a University of Miami approved IACUC protocol. Mice were euthanized when clear signs of morbidity were observed, including lethargy, loss of weight, dehydration. The time to mortality was defined as the time to euthanasia.

### Statistical methods

Data were statistically analyzed using the Student’s *t* test for comparisons between two groups and the Multiple Student’s *t* test when comparing multiple groups using GraphPad Prism 10.0. The results were expressed as mean ± standard error. Tumor volumes were determined by a linear regression [25] utilizing mean ROIs as pseudo-tumor volumes and a slope coefficient was calculated in GraphPad Prism 10.0. A *P* value < 0.05 was considered statistically significant.

## Results

### A Notch signature is present in melanoma brain metastasis

Several studies have shown that main driver gene mutations (e.g., *BRAF*^*V600*^*, NRAS*^*Q61*^), are expressed with similar frequency, or slightly increased frequency, in melanoma brain metastases (MBM) versus extracranial melanoma [26, 27], suggesting brain metastases retain the signature of the tumor of origin.

**Fig. 1:**
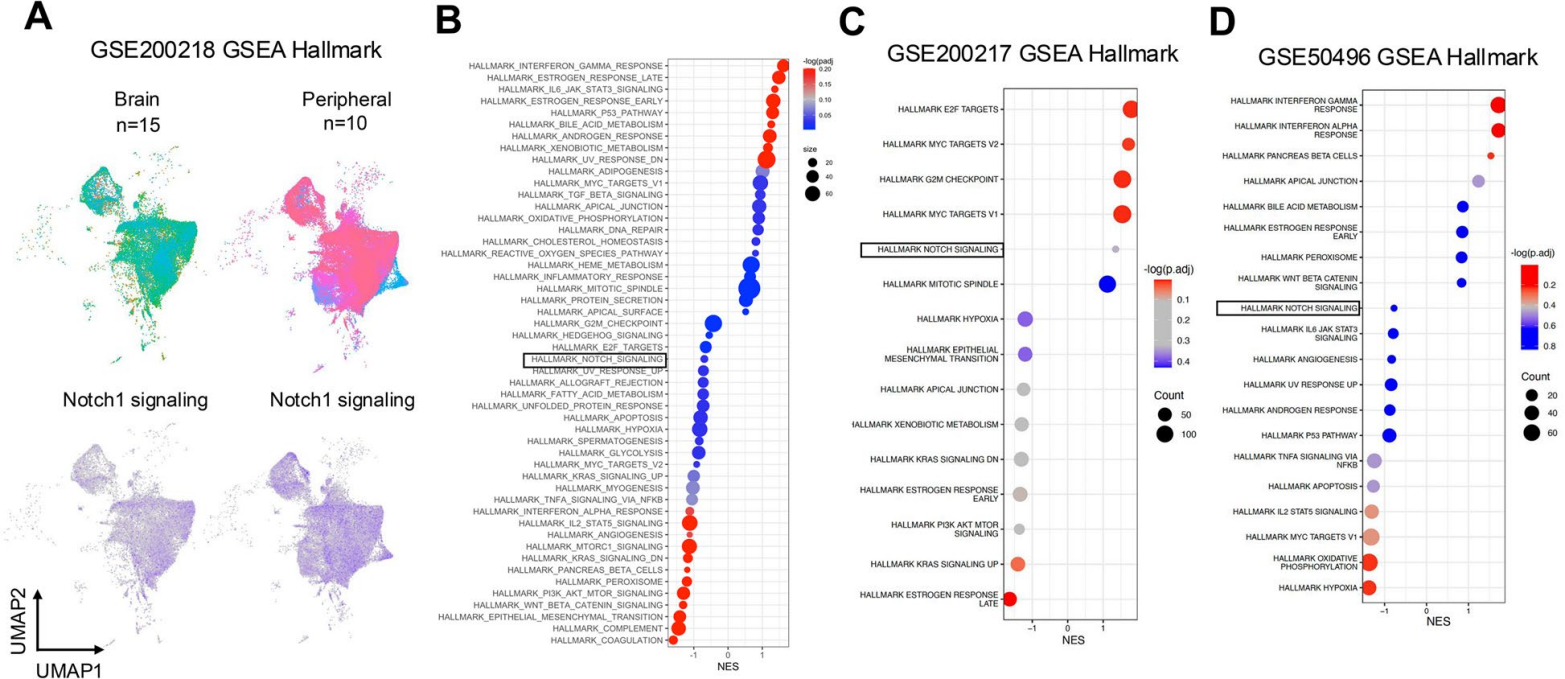
A Notch signature is present in MBMs. **A**-**B** GSE200218: (**A**) Uniform Manifold Approximation and Projection (UMAP) showing the combined tumor cell cluster of melanoma brain (*n* = 15) and peripheral (*n* = 10) metastases (top), with Notch1 signature distribution among the two (bottom). **B** Bubble plot depicting normalized enrichment score (NES) for several Hallmark gene sets from the molecular signature databases (MSigDB) in brain tumor cell cluster compared to the peripheral metastases. **C** Bubble plot depicting normalized enrichment score (NES) for several Hallmark gene sets from the molecular signature databases (MSigDB) in GSE200217 in MBM compared to extracranial metastases. **D** Bubble plot depicting normalized enrichment score (NES) for several Hallmark gene sets from the molecular signature databases (MSigDB) for GSE50496

Notch1 signaling is up-regulated in over 60% of cutaneous melanomas where it is involved in several hallmarks of cancer including growth, progression, survival [8–13]. We recently demonstrated that Notch1 blockade with a neutralizing antibody we developed, led to reduced cutaneous melanoma cell growth and survival and importantly, reduced tumor growth, and enhanced responses to anti-PD1 [15]. However, whether a therapy targeting Notch1 would be beneficial to patients with MBM remains unclear. We therefore first asked whether Notch signaling is retained in MBMs and is therefore a targetable pathway. Several publicly available RNAseq and single nuclei (sn) RNAseq data sets containing MBM and extracranial melanoma lesions were interrogated against a Notch signature (https://www.gsea-msigdb.org/gsea/msigdb/human/geneset/HALLMARK_NOTCH_SIGNALING.html). Gene set enrichment analysis against the MSigDB (Molecular Signatures Database) Hallmark database was performed for all data sets. The data set GSE200218, containing 15 MBMs and 10 peripheral treatment-naïve melanoma metastases analyzed via snRNA sequencing [28], showed no significant difference in the Notch signature between brain and non-brain metastases (Fig. 1A, B and Suppl. Fig. 1A). Similarly, bulk RNAseq data (GSE200217) performed on 24 patient-derived cell lines (in triplicates) corresponding to 4 MBM and 4 peripheral metastases [28] demonstrated no significant difference (Fig. 1C). In a study with 6 matched MBMs (*n* = 6) and extracranial metastases (*n* = 6) for each patient, no significant difference was observed in the Notch signature between groups (Fig. 1D) [27]. To further confirm these data, several genes in the Notch signature were analyzed from the last dataset, including Notch1, Notch2, various Notch canonical targets in the HES and HEY family members of transcription factors, and ligands (Delta-like 1 and Jagged-1). None showed any difference in expression between MBM and extracranial lesions, except for HEY1, which was higher in MBM (Suppl. Fig. 1B). Finally, in a study comparing brain metastases from different tumors of origin (lung, melanoma, renal and breast) [29], no significant difference in the Notch signature was observed among one another (Suppl. Fig. 1C).

Overall, these data show Notch signaling is similarly expressed in both MBM and peripheral lesions, as well as among several brain metastasis of different origin, highlighting the retention of the pathway and its potential significance in MBM pathobiology.

### Notch1 blockade reduces histone availability resulting in replication stress and DNA damage

**Fig. 2:**
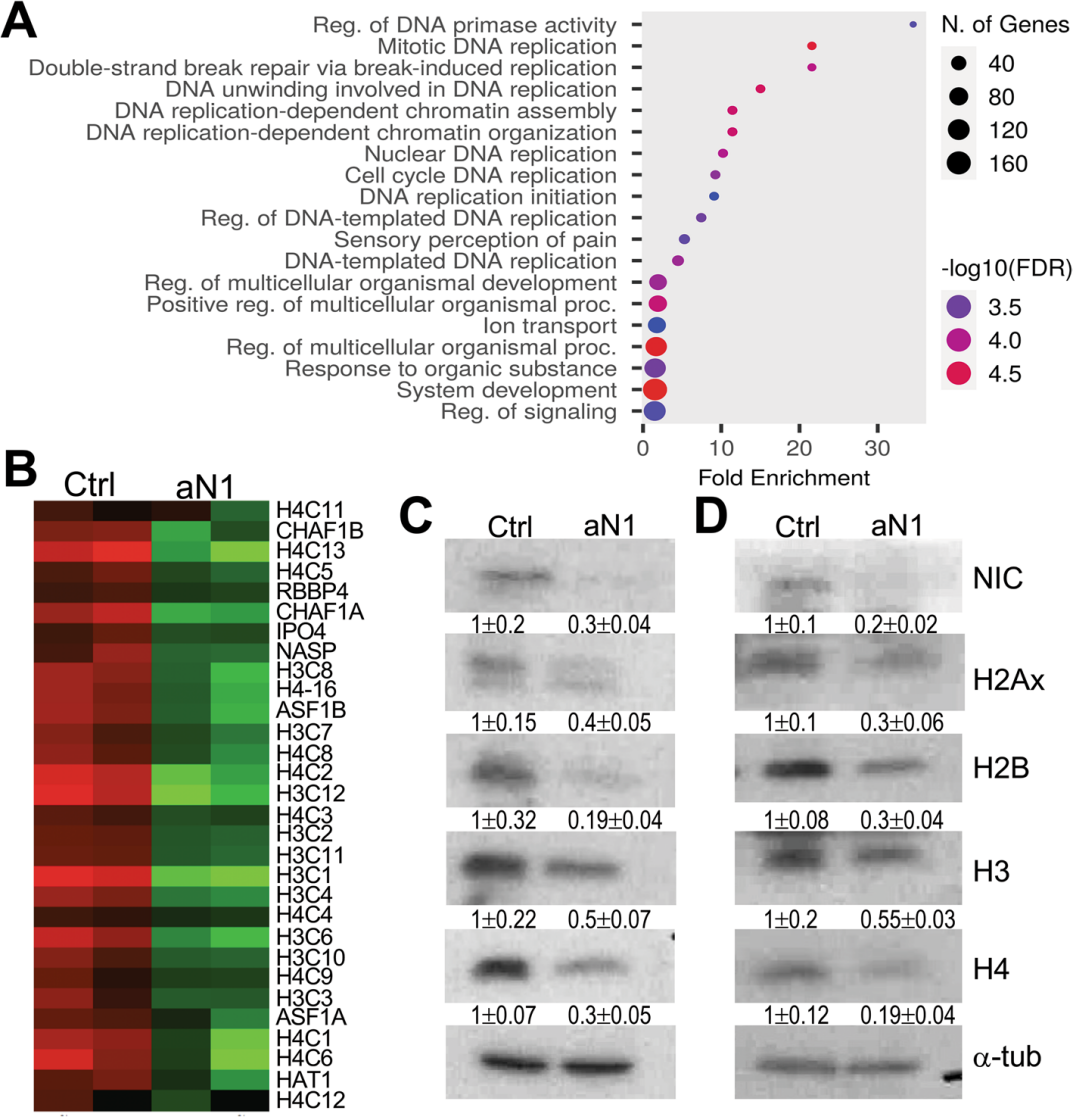
Notch1 blockade affects histone expression. **A** Bubble plot depicting the most significantly inhibited pathways in K457 melanoma cells treated with 50ug/ml anti-Notch1 for 48 h. **B** Heat Map depicting histone mRNAs significantly downregulated in the cells from A. **C**-**D** Core histone protein levels in K457 (**C**) and A375 (**D**) cells treated as in A. α-tubulin was used as loading control. Band intensity is the mean between two experimental repeats for each cell line, normalized to the loading control for each lane. Control was then set at 1 and the levels in aN1 treated cells normalized to ctrl

To investigate further the mechanisms of Notch1 in melanoma, we performed RNAseq on K457 melanoma cells treated with IgG control or anti-N1 for 48 h. Surprisingly, gene set enrichment analysis revealed the most significantly inhibited pathways were DNA replication and chromatin and nucleosome organization and assembly (Fig. 2A), with several histone genes significantly inhibited (Fig. 2B). To validate the RNAseq data, protein lysates from K457 and A375 cells treated with anti-N1 were analyzed for core histone levels. Blockade of Notch1 activation resulted in 50 to 80% reduction of core histones levels in both lines (Fig. 2C, D). Intriguingly, gene set enrichment analysis of microarray data of WM266-4 melanoma cells expressing shGFP or shNotch1 [15], revealed that the most significantly downregulated pathways are involved in cell cycle, mitosis and chromosome organization, with several histone genes downregulated by Notch1 inhibition (Suppl. Fig. 2), further supporting a role of Notch1 in histone regulation and corroborating the results obtained with pharmacological blockade of Notch1 by anti-N1.

**Fig. 3:**
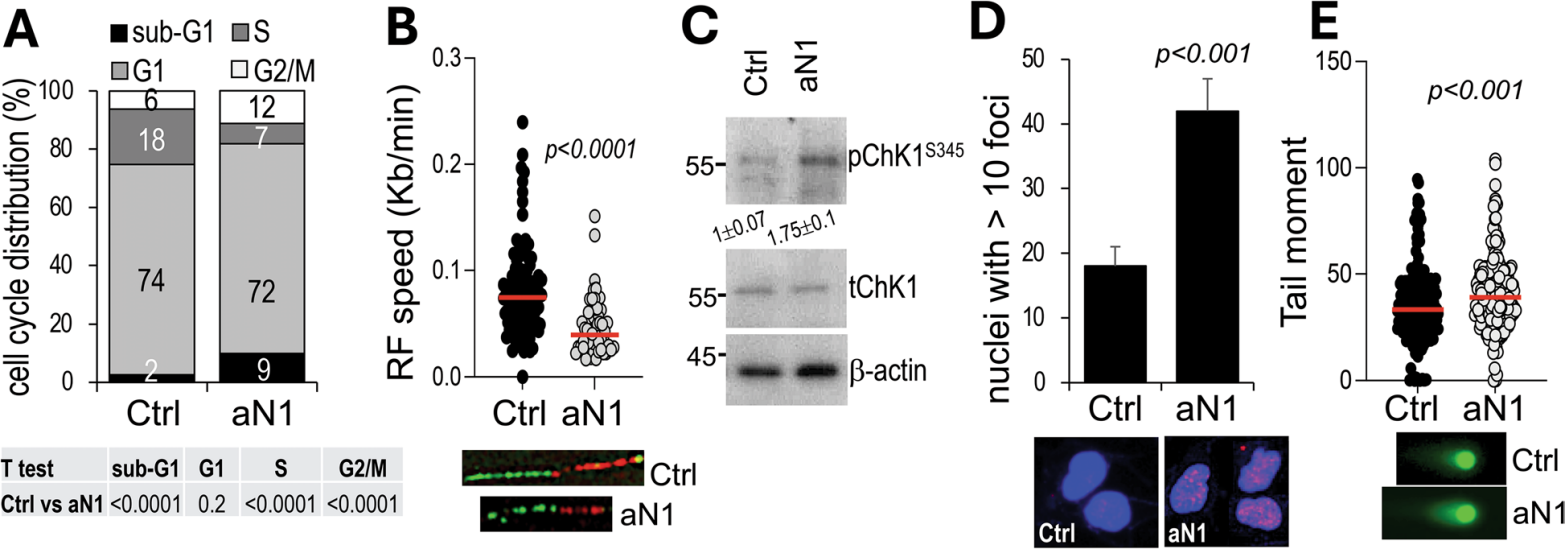
Notch1 blockade causes replication stress and DNA damage.** A** % of K457 cells in each phase of the cell cycle at 48 h treatment with anti-N1 (100ug/ml). **B** DNA replication fork speed in K457 cells. 100 fibers were analyzed for each group. Speed: IdU (red) tract length/time of the IdU pulse (30 min) = um/min. um were converted to Kilobases (Kb) using the conversion factor 1um = 2.59 kb. **C** pChK1^S345^ is induced because of RF stress and ATR activation. Band intensity is the mean between two experimental repeats normalized to the loading control for each lane. Control was then set at 1 and the levels in aN1 treated cells normalized to ctrl. **D** γH2 AX foci in K457 cells treated as in A. n ≥ 50 nuclei per section were counted for 5 section per group. **E** Neutral Comet Assay of K457 cells treated as in A. n ≥ 200 comets/group. Significance of the differences among groups was determined by the Student’s *t* test. Data are the mean of three independent experiments each performed in triplicate

To accommodate DNA replication in S phase, histone proteins must double in number to maintain the proper compaction and organization of genomic DNA, hence, cells synthesize new histones in late G1 and S phase [30]. If histones are not sufficient, cells cannot complete mitosis and accumulate in G2/M [31], while undergoing DNA replication stress. A cell cycle analysis in K457 cells treated for 48 h with anti-N1 indeed revealed that Notch1 blockade increased by 2-folds the percentage of cells in G2/M (12% vs 6% in anti-N1 and control cells, respectively), while causing a 2.5-fold reduction of cells in S phase (18% vs 7% in anti-N1 and control cells, respectively—Fig. 3A). Similar data were observed in A375 cells in which anti-N1 also caused a 2.5 fold increase in cells accumulating in G2M and an almost threefold reduction in cells in S phase (Suppl. Fig. 3A). Cell cycle data with standard deviation for both A375 and K457 cells are shown in Suppl. Fig. 4A, B. Representative flow cytometry profiles are shown in Suppl. Fig. 4C, D.

The accumulation of cells in G2/M when Notch1 activity is inhibited, suggests cells experience replication stress, likely due to the reduced histones, as impaired histone biosynthesis results in slower replication fork (RF) progression [32]. We therefore tested if blockade of Notch1 would result in the slowing of RFs. We observed a twofold reduction in RF speed in K457 cells (Fig. 3B), which was measured by incubating cells first with CldU (chloro- deoxyuridine) for 30 min, and then by pulsing them with IdU (Iodo-deoxyuridine) for the same time. The IdU tracts were measured, and the length divided by the IdU pulse time (30 min) and converted in Kb/min using the conversion factor: 1um = 2.59 Kb [33].

The normal response of cells undergoing RF stress, is the activation of a DNA Damage Response (DDR) via ATR/ChK1 at Chk1^S345^, to pause cells in the cell cycle and allow fork restart [34]. Indeed, cells treated with anti-N1 activated ChK1^S345^ (Fig. 3C). However, as the stress persists (cells are continuously exposed to anti-N1), cells are unable to repair RFs, which then collapse into double stranded breaks (DSBs) [35]. Cells undergoing anti-N1 treatment show a twofold increase in γ-H2 AX foci (Fig. 3D); and a 20% increase in tail moment of a neutral comet assay, which measures the amount of DSBs specifically [35–37] (Fig. 3E). Confirming these data, A375 cells also showed a twofold reduction in RF speed upon anti-N1 treatment, as well as a twofold increase in γH2 AX foci and a 32% increase in tail moment (Suppl. Fig. 4B, C, D).

In summary, Notch1 blockade causes genomic instability with cells undergoing unresolved replication fork stress which then results in DNA damage.

### Blockade of Notch1 and ChK1 exacerbates genomic instability in melanoma cells

Blockade of Notch1 by anti-N1 causes replication fork stress and DNA damage. Cells also activate a DNA Damage Response (DDR) via ATR/ChK1 to address RF stress and counteract the damage. Thus, we reasoned that cells undergoing Notch1 inhibition would depend heavily on ChK1 activation, and that concomitant blockade of both Notch1 and ChK1 should further increase RF stress and DNA damage.

To validate our assumption, we used prexasertib (prex). Prex is a ChK1/2 inhibitor currently in several phase 2 clinical trials, both as single agent and in combination with chemotherapy, in ovarian cancer [16]; breast cancer and colon cancer [17]; and in a phase 1 trial for pediatric patients with recurrent or refractory brain tumors [18].

**Fig. 4:**
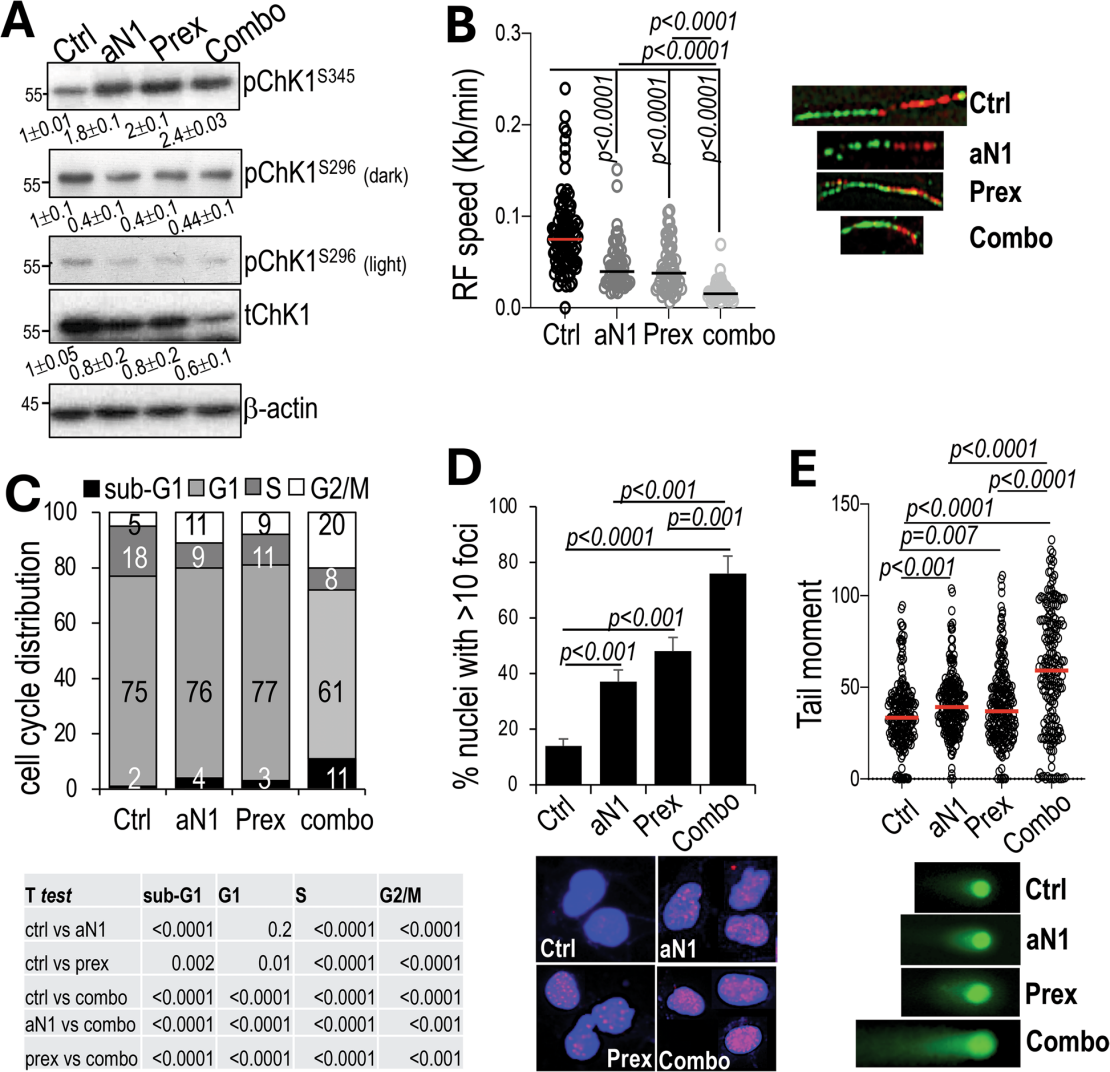
Concurrent blockade of Notch1 and ChK1 increases replication stress and DNA damage. **A** pChK1^S345^, pChK1^S296^ (autophosphorylation site that leads to full activity) and total ChK1 in K457 cells treated with anti-N1 (100ug/ml) and prexasertib (prex) (5 nM), alone or in combination, for 48 h. Band intensity is the mean between two experimental repeats normalized to the loading control for each lane. Control was then set at 1 and the levels in aN1 and prex treated cells normalized to ctrl. **B** DNA replication fork speed. 100 fibers were analyzed for each group. Speed: IdU (red) tract length/time of the IdU pulse (30 min) = um/min. um were converted to Kilobases (Kb) using the conversion factor 1um = 2.59 kb.). Representative DNA tracts are shown on the right. **C** % of K457 cells treated as in A, in each phase of the cell cycle. Significance between treatment for each phase was calculated by the Student’s *t* test (table below). **D** γH2 AX foci in cells treated as in A. n ≥ 50 nuclei per section, 5 section per group. Representative pictures are shown below. **E** Neutral Comet Assay of cells treated as in A. n ≥ 200 comets/group. Representative comets are shown below. Data are the mean of three independent experiments each performed in triplicate. Significance of the differences among groups was determined by the Student’s *t* test

We first tested the activation of ATR/ChK1 under the different treatment conditions. As shown in Fig. 4A, cells treated with anti-N1 or prex or the combination of the two, show activation of Chk1 at Serine 345, indicating response to DNA damage. On the other hand, the autophosphorylation site at serine 296, which leads to full activation, is clearly reduced by prex, indicating efficacy of the inhibitor [24]. Interestingly, Notch1 blockade also reduced ChK1^S296^, suggesting a role of Notch1 in ChK1 activation. Notch1 has been shown to activate ChK1 through ATR in breast cancer [38], thus it is possible that its inhibition may result in reduced activation in melanoma cells. RF speed was significantly reduced by twofold by prex, which also led to twice the cells accumulating in G2/M compared to controls. A threefold increase in γH2 AX foci (Fig. 4B-D) and a 20% increase in tail moment (Fig. 4E) was also observed. These phenotypes resemble those caused by anti-N1 alone.

However, when Notch1 and ChK1 were inhibited simultaneously by anti-N1 and prex, the effects were significantly worsened. RF speed was reduced by fourfold compared to twofold of anti-N1 or prex alone; 20% cells accumulated in G2/M compared to 11% and 9% of anti-N1 and prex, respectively, with a proportional reduction in cells in S phase. DNA damage, in the form of DSBs, was also significantly boosted, with a fourfold increase in γH2 AX foci and a 60% increase in tail moment compared to control. To further validate these data, A375 cells were also treated with anti-N1 and prex, either alone or in combination (Suppl. Fig. 3). Again, both treatments resulted in the induction of ChK1 phosphorylation at ser345, indicating activation of the pathway due to DNA damage response; and both treatments led to reduce phospho ChK1^S296^, indicating blockade of the kinase (Suppl. Fig. 3E). RF speed was reduced by fourfold by the combination treatment (Suppl. Fig. 3B), which likely resulted in 19% cells accumulating in G2/M (Suppl. Fig. 3A). The percentage of cells with > 10 nuclei with γH2 AX positive foci was increased by fourfold; while the tail moment was increased by 45% over control (Suppl. Fig. 3C, D).

These results support the notion that blockade of Notch1 creates a dependency on the repair pathway ATR/ChK1, thus creating a new vulnerability, that could be exploited therapeutically.

### Blockade of Notch1 and ChK1 causes mitotic catastrophe and cells death

Notch1 and ChK1 blockade, particularly if inhibited simultaneously, leads to RF stress and consequent unresolved DNA damage. We therefore sought to determine if mitotic catastrophe was a mechanism contributing to melanoma cell death. Mitotic catastrophe is a mechanism of cell death that prevents the survival of cells that are incapable of completing mitosis because of defects of the mitotic apparatus, DNA damage, and prolonged and unresolved replication stress [39, 40].

**Fig. 5:**
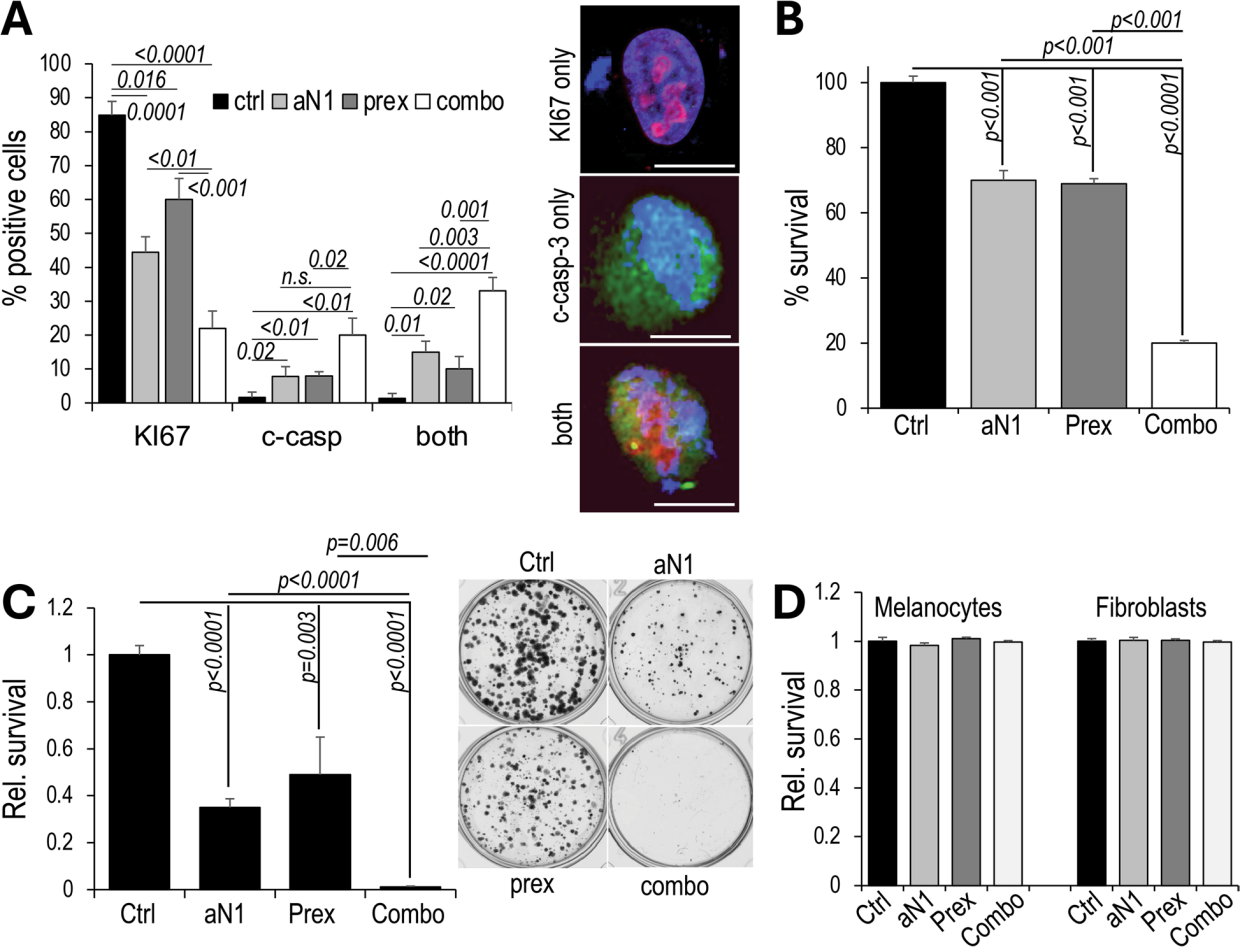
Concurrent blockade of Notch1 and ChK1 causes mitotic catastrophe and cell death.** A** % of A375 cells positive for cleaved-caspase-3 (apoptotic), KI67 (proliferative) or double positive (mitotic catastrophe) after treatment with anti-N1 (100ug/ml) and prexasertib (5 nM), alone or in combination, for 24 h. Representative staining of each condition is shown on the right (scale bar: 10um). **B** Survival of A375 at 48 h post treatment determined by trypan blue staining. **C** Clonogenic assay of A375 treated with anti-N1, prex and a combination of the two. A picture of representative colonies for each condition is shown on the right. **D** normal human melanocytes and human fibroblasts treated as the cells in B. Statistical differences were determined by the Student’s *t* test. Data are the mean of three independent experiments each performed in triplicate

A375 cells were treated with either inhibitor alone or in combination for 24 h and then stained with DAPI, to identify the nuclei. anti-cleaved-caspase-3 and anti-KI67 were used to identify apoptotic and proliferating cells, respectively. Cells that are doubled positive are cells experiencing mitotic catastrophe [41]. We found that concomitant blockade of Notch1 and ChK1 leads to a significant increase in cells experiencing mitotic catastrophe, as shown by 30% double positive cells in the anti-N1/prex treated group compared to 2% controls (Fig. 5A). K457 cells treated with anti-N1 and prex for up to 72 h showed similar results (Suppl. Fig. 5A). In this case, cells were stained with anti-cleaved-caspase-3 to identify apoptotic cells, and anti-phospho-Histone H3, to identify proliferating cells. Starting at 24 h, the cells treated with the combination anti-N1/prex showed the highest level of mitotic catastrophe that was maintained throughout the tested time points. Suppl. Fig. 5B, C shows single and double positive cells distribution at 24 h, and representative gating strategy used to identify single and double stained cells, respectively.

The extreme stress melanoma cells experience while undergoing blockade of Notch1 and ChK1, is likely to lead to cell death. Indeed, A375 melanoma cell survival, measured by trypan blue staining, was reduced by 30% in cells treated with anti-N1 or prex, at 48 h, while the combination led to 80% reduced cell survival (Fig. 5B). K457 cells were affected similarly by the treatment combination (Suppl. Fig. 5D). In a colony survival assay in which the remaining colonies were counted after 12 days in culture, the number of colonies was reduced by 60% in cells treated with either anti-N1 or prex, however, the combination of the two led to virtual elimination of melanoma colonies in culture (Fig. 5C). The combination treatment appears to be synergistic (Suppl. Fig. 6), particularly at the higher concentrations of each drug, in both cell lines. Synergy was determined using the free software SiCoDEA (https://sicodea.shinyapps.io/shiny/) [42]. Notably, normal human melanocytes and fibroblasts were not at all affected (Fig. 5D), indicating potential safety of a treatment regimen combining anti-N1 and prex, and indicating melanoma cells may be likely addicted to these two pathways for survival and therefore highly sensitive to their inhibition.

### Concurrent inhibition of Notch1 and ChK1 increases survival of animals bearing MBMs

The in vitro data strongly support the blockade of both Notch1 and ChK1 as anti-cancer approach, particularly in MBM where the standard of care is radiation therapy which acts by causing DNA damage and cell death. We therefore wanted to address the utility and feasibility of our new targeted therapy in MBM.

**Fig. 6:**
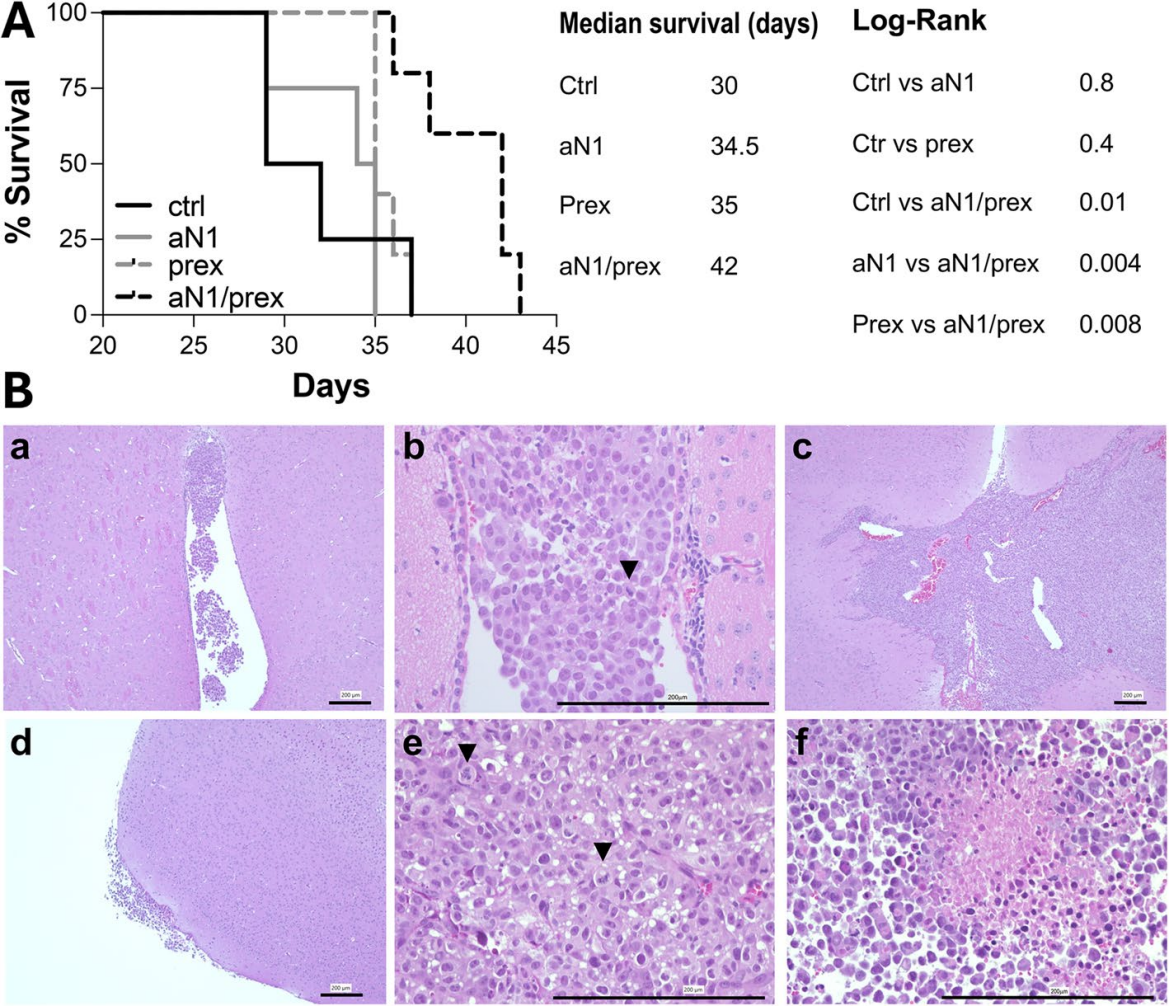
Concurrent blockade of Notch1 and ChK1 improves the survival of animals bearing MBMs. **A** 10^5^ A375 cells expressing luciferase, were inoculated stereotactically into the right cerebral cortex of athymic nude mice. Tumors were monitored twice weekly since inoculation. Kaplan–Meier curves were generated with Graph Pad Prism10. Logrank p values, median overall survival (OS) and Hazard Ratio (HR) are shown. **B** Representative H&E staining. Scale bar: 200um

10^5^ A375 melanoma cells labeled with luciferase, were stereotactically implanted in the brain of athymic nude mice, as previously shown [20]. The day after, mice were distributed in 4 groups of treatment based on the radiance intensity (ROI) of each tumor so that each group would contain tumors emitting similar radiance, as an indirect measurement of tumor mass (Suppl. Fig. 7A). While prexasertib has been shown to cross the blood brain barrier (BBB) [43], prior to treatment with anti-N1 (100 mg/Kg), we performed a qualitative assay described by *Rao *et al. [44], to determine whether anti-N1 would cross the BBB and reach the tumor. The antibody was labelled with Alexa fluor-647 and then inoculated I.P. The fluorescence intensity was then analyzed at 3, 24, 48 and 72 h post inoculation in parallel with luminescence (Suppl. Fig. 7B, C). The antibody is stable and persists in the tumor for at least 24 h, after which a slight decline in fluorescence intensity is observed. Thus, we opted for a daily dose of anti-N1 delivered intraperitoneally (I.P), at 100 mg/Kg, while prex was delivered subcutaneously (s.c.), twice a day for 3 consecutive days, followed by 4 days holiday period as previously described [24].). Tumor growth was followed over time and estimated by a linear regression [25] utilizing mean ROIs as pseudo-tumor volumes. The single treatment with anti-N1 or prex slowed down the growth, with the combination leading to further growth inhibition (Suppl. Fig. 7D). This likely led to a ~ 30% increase in survival in the treatment combination group compared to control. Single treatment with either anti-N1 or prex led to a slight, albeit not significant, increase in survival (~ 14%) (Fig. 6A). At the time of tumor collection, i.e. when mice were euthanized at the end time point due to morbidity, all tumors in all groups appear similarly invasive, with cells spilling in the ventricles, invading the brain parenchyma and clustering at the leptomeninges (Fig. 6B a, b, c, d** –** representative images from control tumors). Tumor cells in all groups also show numerous mitotic figures and areas of necrosis indicating aggressiveness (Fig. 6B e, f** –** representative images from control tumors).

In summary, concomitant blockade of Notch1 and ChK1 significantly reduces brain metastatic burden leading to increased survival.

## Discussion

Patients with MBMs have experienced an increase in survival due to the advent of targeted and immunotherapy. However, responses are short lived for targeted therapy and only half of the patients respond to immunotherapy. Radiation therapy remains the gold standard for the treatment of brain metastases. Unfortunately, melanoma cells are quite resistant to radiation, which decreases the efficacy of radiotherapy. These limitations in treatment leave therefore room for improvement.

DNA replication is a fundamental cellular process in which the entire genome is duplicated once during the cell cycle S phase; and accurate DNA replication is essential for the faithful transmission of genetic information through cell divisions to ensure genomic stability [45].

DNA Replication stress (RS) can be caused by both endogenous (i.e., alternative structures of DNA, centromeres, telomeres, DNA binding non-histones, ROS, transcription conflicts) and exogenous (i.e., DNA damage caused by UV, radiation or genotoxic and cytotoxic substances, nucleotide loss, oncogene induced abnormal proliferation) stresses [46, 47]. RS manifests as a slowing and/or stalling of replication forks (RFs), and it is a major driver of genome instability and a hallmark of cancer cells. Indeed, RS is selectively higher in cancer cells than in normal cells, due to their inherent genomic instability that drives tumorigenesis, which makes cancer cells more dependent on RS response pathways for survival. Disruption of these defense mechanisms further enhances the RS state, allowing cell cycle progression despite high levels of unrepaired DNA damage and accumulation of stalled replication forks in the genome, which trigger mitotic catastrophe and apoptosis [48].

Here we show that Notch1 is expressed in melanoma brain metastasis and that, given its role in all hallmarks of melanoma, can be a new therapeutic target for MBMs. By using our selective anti-Notch1 neutralizing antibody previously described [15], we discovered that Notch1 blockade causes genomic instability by reducing histone availability, leading to replication stress and DNA damage. Naturally, cells activate the DNA Damage Response pathway ATR/ChK1 to counteract the damage, creating a new vulnerability that can be exploited therapeutically. Indeed, we show that targeting both Notch1 and ChK1 maximizes the damage leading to mitotic catastrophe and cell death and importantly, significantly increases survival of mice bearing melanoma MBMs.

The interdependency of Notch1 and ATR/ChK1 pathways and the sensitivity of melanoma cells to their concurrent inhibition, is comparable to the synthetic lethal interactions observed for example in BRCA mutated tumor cells and PARP (poly (ADP-ribose) polymerase) inhibitors (PARPi). BRCA mutated cancer cells, when treated with PARPi cannot repair DNA neither via Homologous Recombination (BRCA dependent) nor via Base Excision Repair (PARP1 dependent), which results in cell death [49]. Similarly, Notch1 blockade causes RS and a critical dependency to the ATR/ChK1 repair pathway which leads to cell death when both are concurrently inhibited.

In summary, Notch1 and ChK1 blockade may represent a new treatment approach, alone or better yet, in combination with targeted, immuno- and radiotherapy in the treatment of melanoma brain lesions.

## Supplementary Information

Supplementary Material 1

## Data Availability

The data generated during the current study are available from the corresponding author on reasonable request.
