# Supplementary material for: Co-inhibition of Notch1 and ChK1 triggers genomic instability and melanoma cell death increasing the lifespan of mice bearing melanoma brain metastasis
Source: J Exp Clin Cancer Res. 2025 May 28;44:163. doi: 10.1186/s13046-025-03411-w (PMC12117938; doi:10.1186/s13046-025-03411-w)
Supplement: Supplementary file 1 — Supplementary Material 1 [file 13046_2025_3411_MOESM1_ESM.pdf]

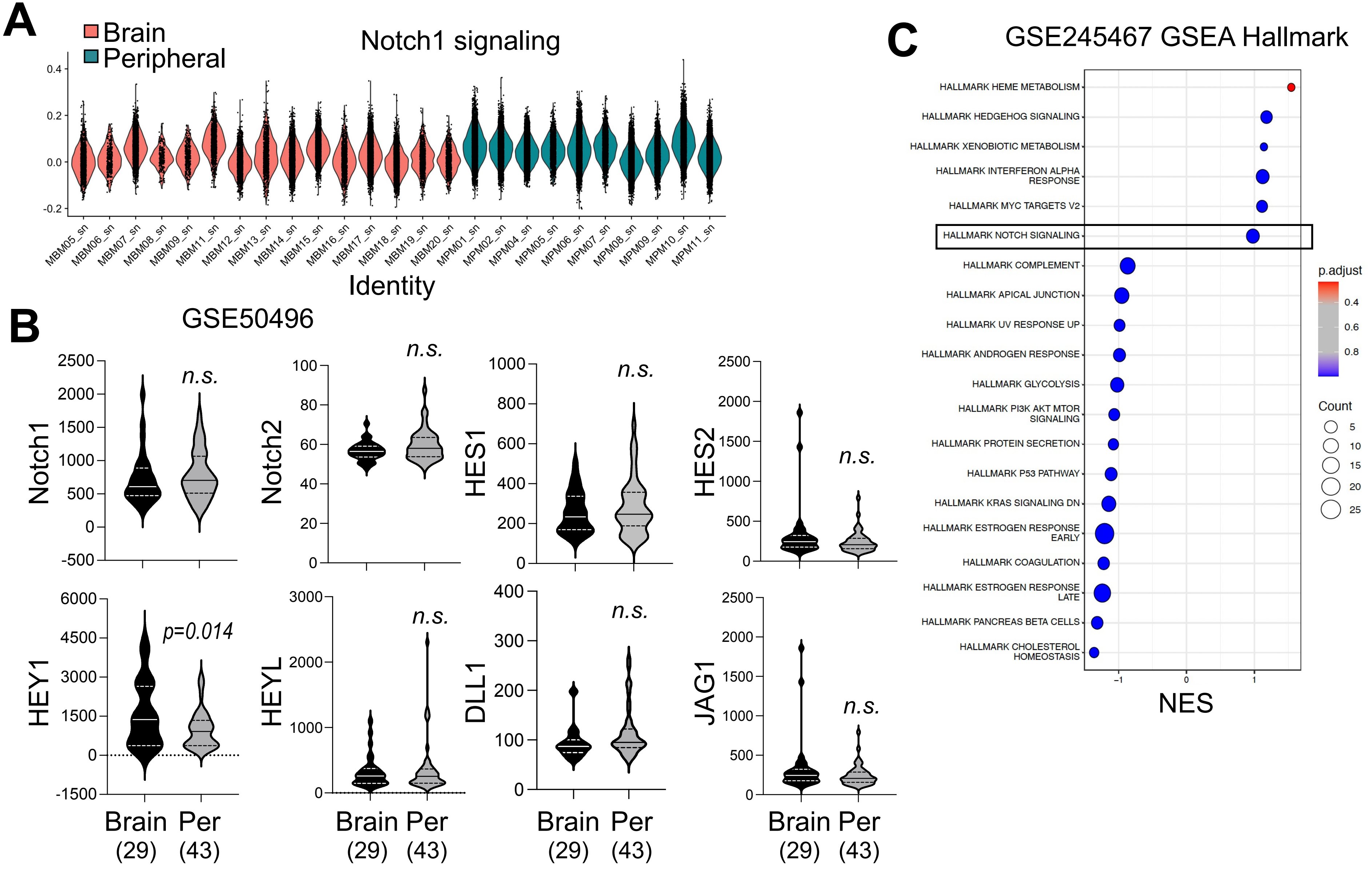

Suppl. Fig. 1: A) violin plot representing relative expression levels of a Notch1 signature in each sample in brain and peripheral metastasis (GSE200218). **B)** Violin plots depicting relative mRNA expression of several Notch pathway genes in brain and peripheral melanoma metastases (GSE50496). **C)** Bubble plot depicting normalized enrichment score (NES) for several Hallmark gene sets from the molecular signature databases (MSigDB) for GSE245467.

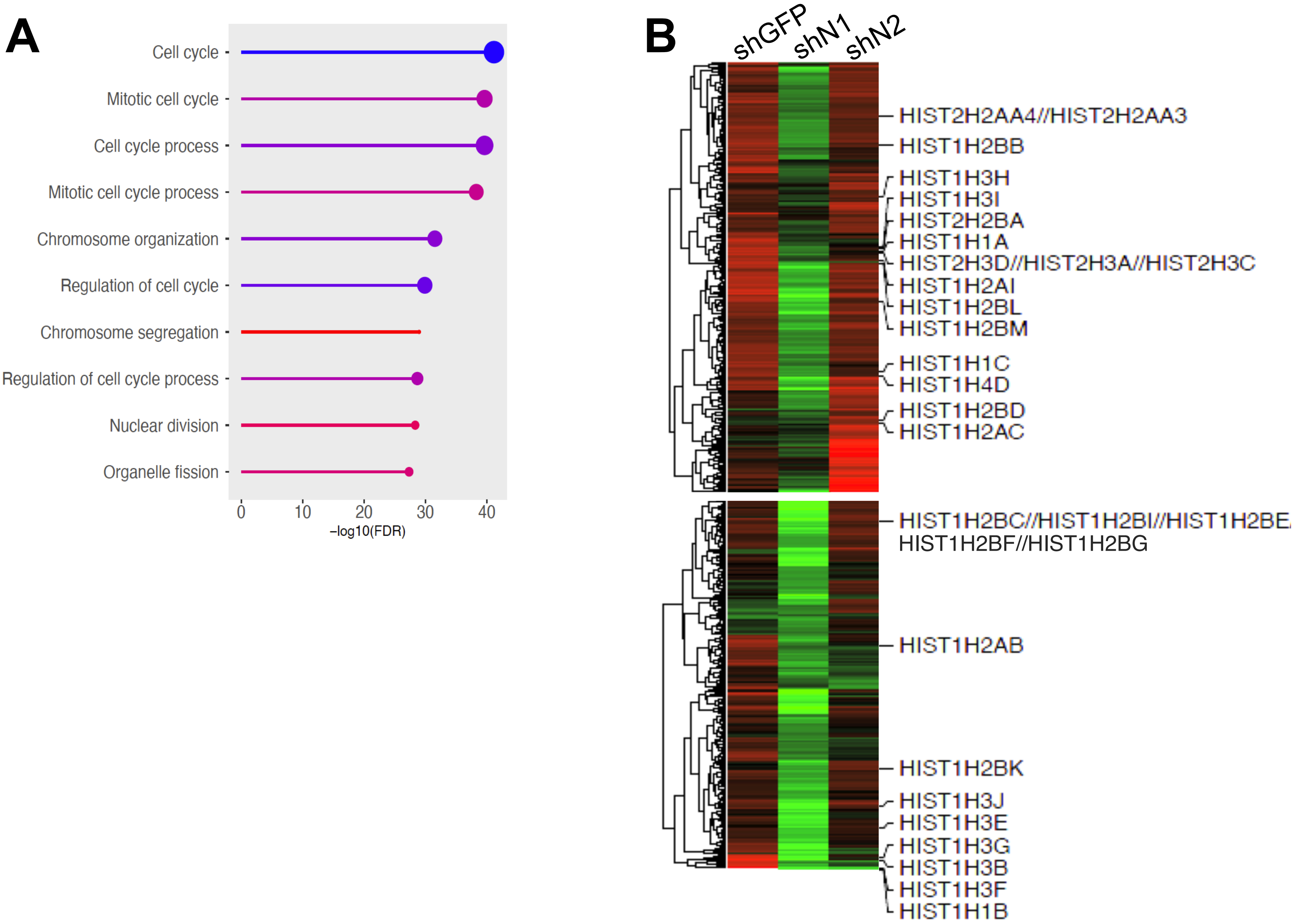

Suppl. Fig. 2: A) Bubble plot depicting the most significantly inhibited pathways in WM266-4 melanoma cells expressing shGFP or shRNA against Notch1 and 2 (Ref# 15). **B)** Heat Map depicting histone mRNAs significantly downregulated in the cells from A. Note this is the old nomenclature for histone genes.

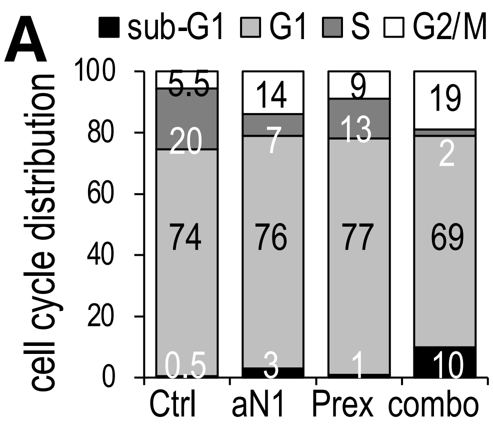

T test	sub-G1	G1	S	G2/M
ctrl vs aN1	<0.0001	0.006	<0.0001	<0.0001
ctrl vs prex	0.005	0.0002	<0.0001	<0.0001
ctrl vs combo	<0.0001	<0.0001	<0.0001	<0.0001
aN1 vs combo	<0.01	0.006	<0.001	<0.001
prex vs combo	<0.001	<0.001	<0.0001	<0.001

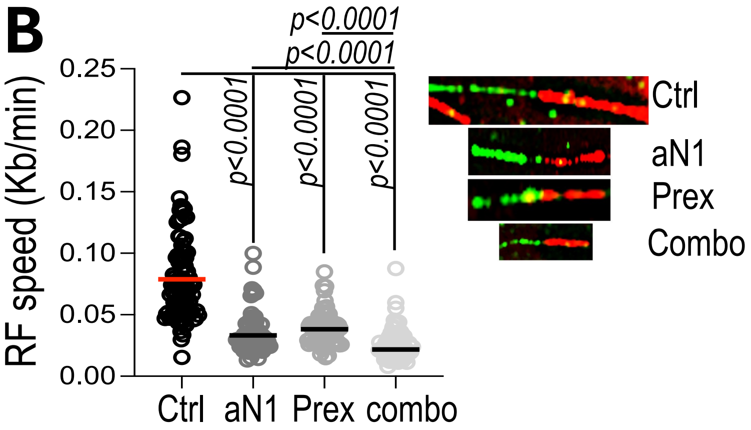

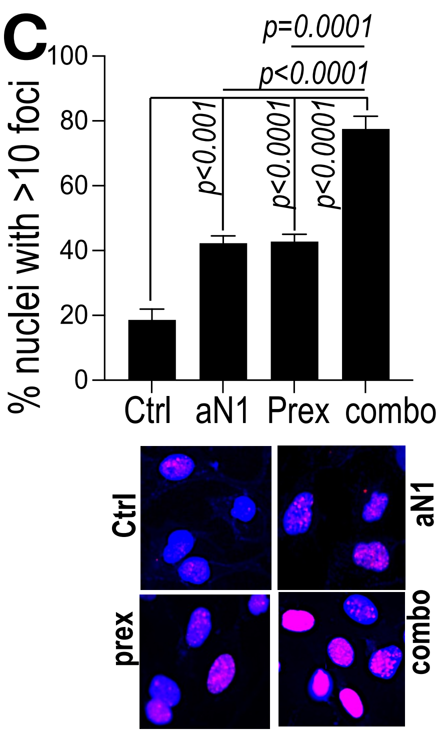

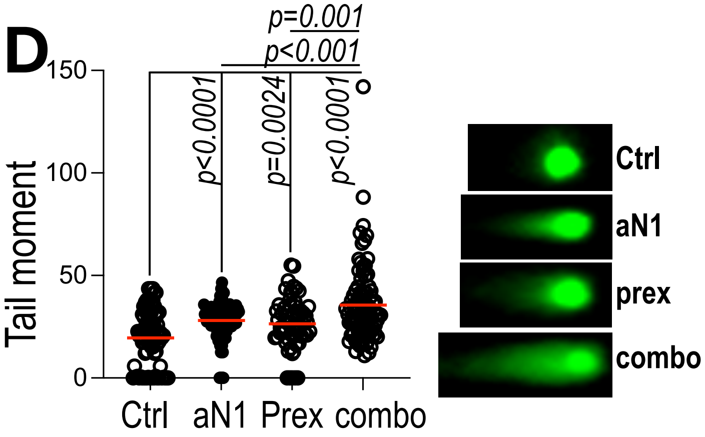

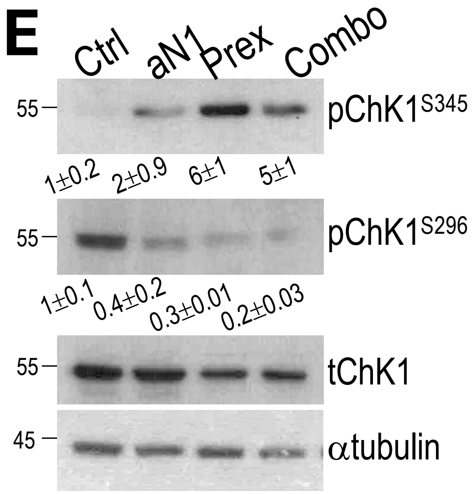

Suppl. Fig. 3: A) % of A375 cells in each phase of the cell cycle at 48 hours treatment with anti-N1 (50ug/ml) and prexasertib (5nM). **B)** DNA replication fork speed. 100 fibers were analyzed for each group. Speed: IdU (red) tract length/ time of the IdU pulse (30 min)= um/min, then converted to Kb/min. Representative DNA tracts are shown. **C)** γH2AX foci in cells treated as in A. n≥50 nuclei per section, 5 section per group. Representative pictures are shown. **D)** Neutral Comet Assay of cells treated as in A. n≥200 comets/group. Representative comets are shown. **E)** pChK1^{S345}, pChK1^{S296} and total ChK1 in A375 cells treated as in A. Band intensity is the mean between two experimental repeats normalized to the loading control for each lane. Control was set at 1 and the levels in aN1, prex and combo treated cells normalized to ctrl. All other data are the mean of three independent experiments each performed in triplicate.

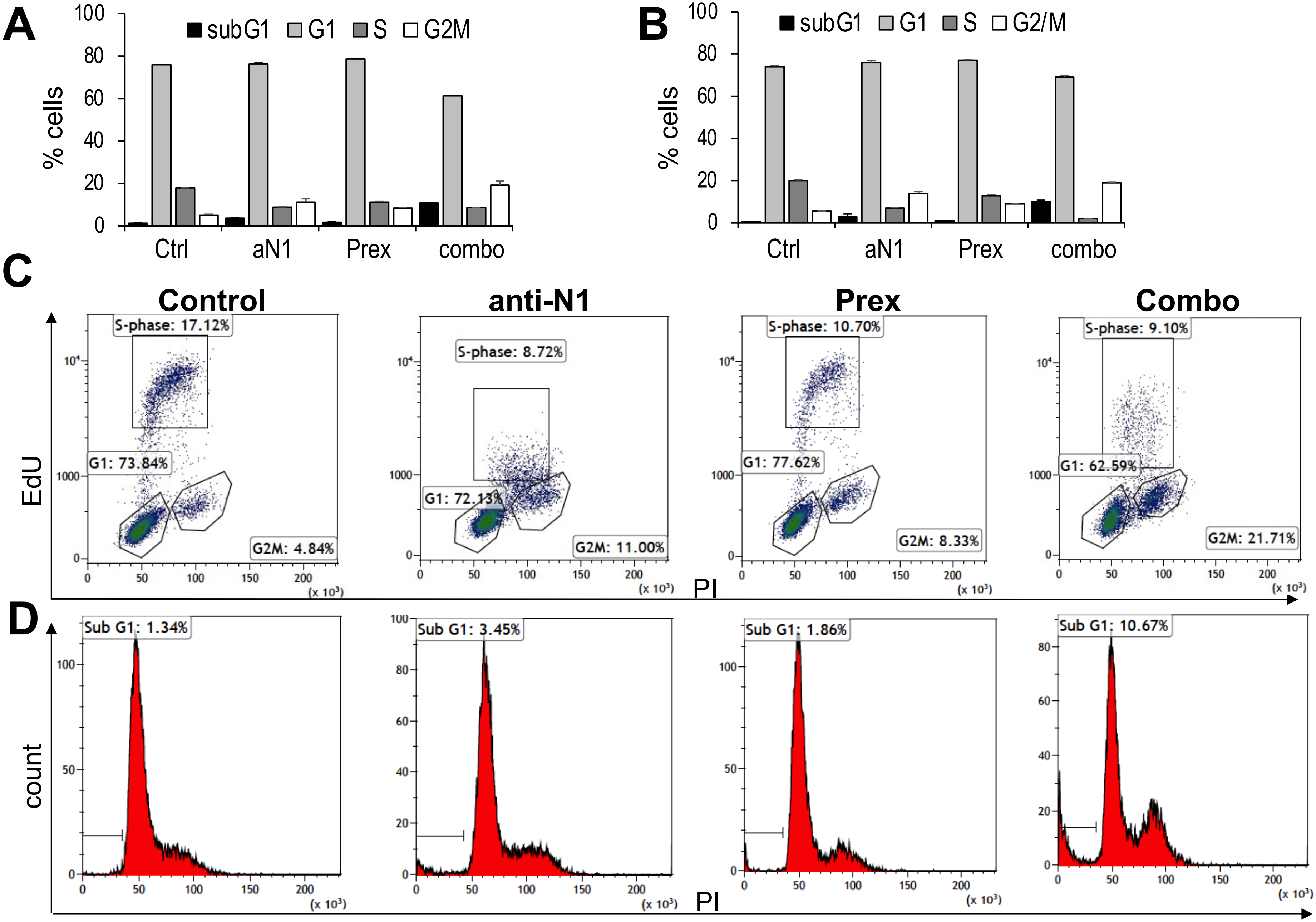

Suppl. Fig. 4: A, B) cell distribution among phases of A375 (A) and K457 (B), with SD values. Statistical analysis is shown in Fig. 3 for K457 and Suppl. Fig. 3 for A375. **C)** Representative cell cycle gating strategy for each treatment group. **D)** representative gating strategy to identify sub-G1 cells.

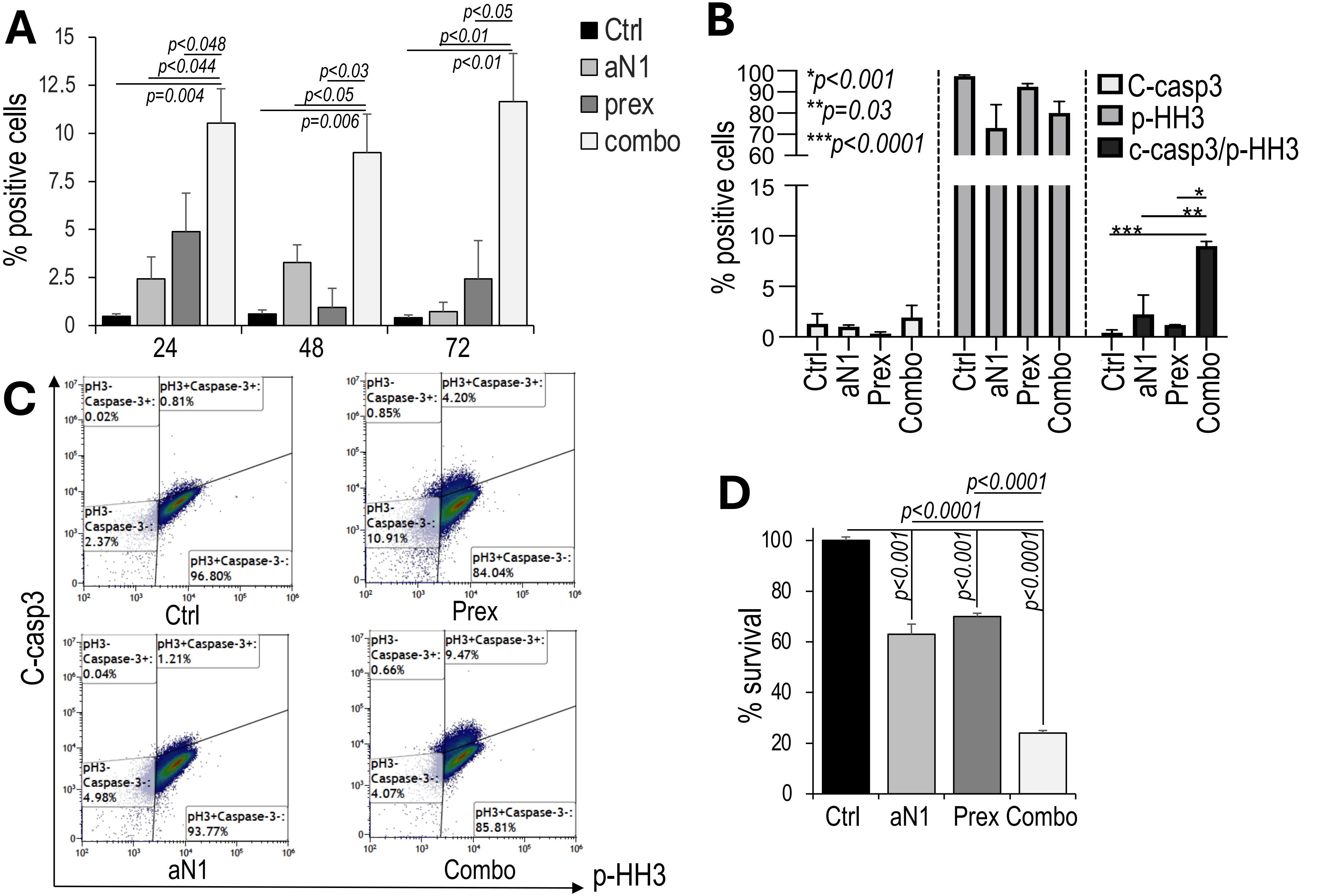

Suppl. Fig. 5: A) % K457 melanoma cells showing dual positivity for cleaved caspase-3 (apoptosis) and phospho-histone H3 (proliferation) quantified via flow cytometry, after 24, 48 and 72 hour treatment with aN1 and prex. **B)** % K457 melanoma cells showing positivity for cleaved caspase 3 and phospho-histone H3, or with dual staining at 24 hours. **C)** representative gating strategy used to identify single and double stained cells. **D)** % survival of K457 cells treated for 48 hours with aN1 (100ug/ml), prexasertib (5nM) or a combination of the two (trypan blue).

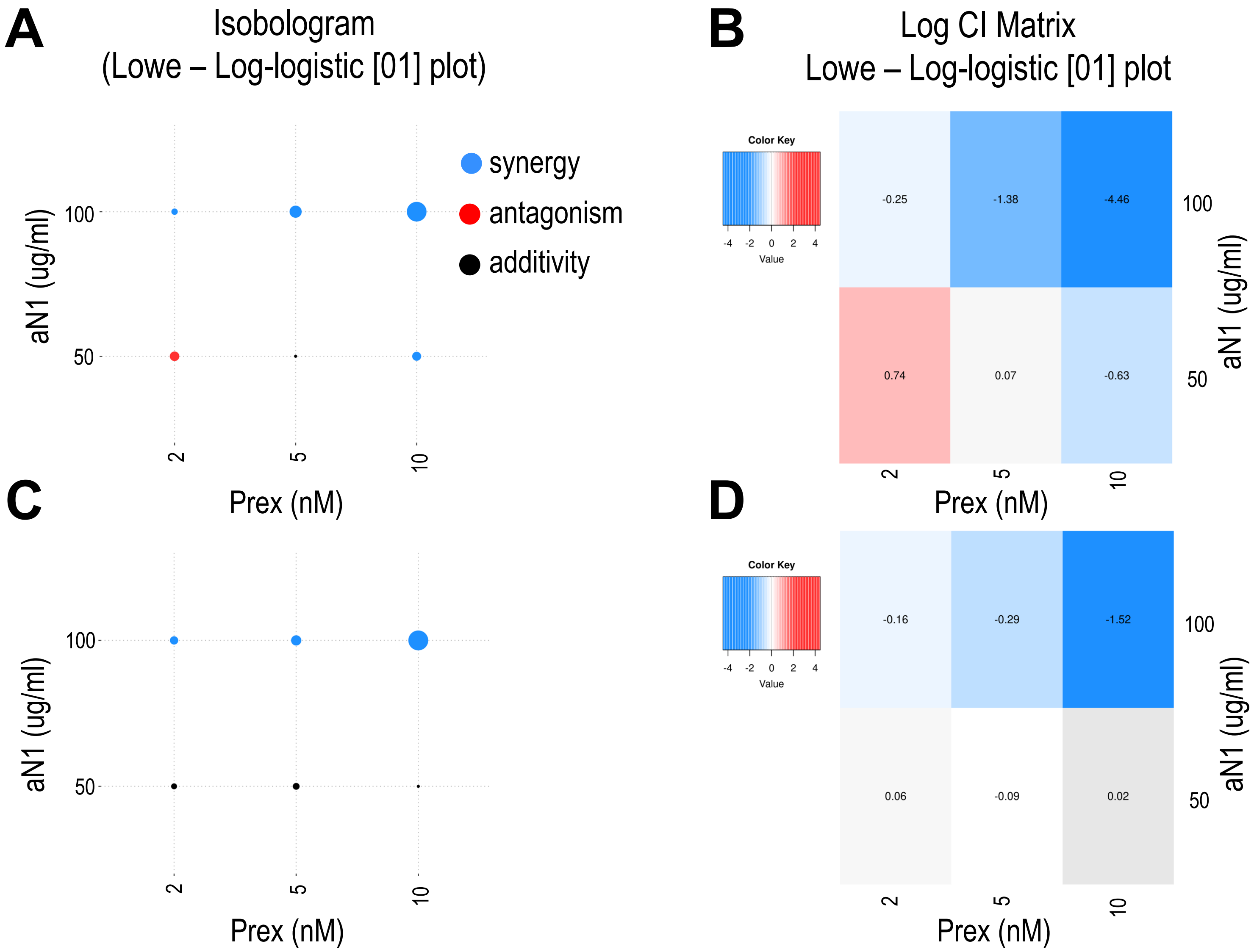

Figure 6: Combination index (CI) values calculated for K457 cells (**A, B**) and A375 cells (**C, D**) treated with anti-N1 and prex combinations for 48 h. The higher the size of the circle, the higher the CI power. For both lines, the highest concentrations result in the highest synergy. Combination indexes were determine using the free software SiCoDEA (https://sicodea.shinyapps.io/shiny/ - REF#42).

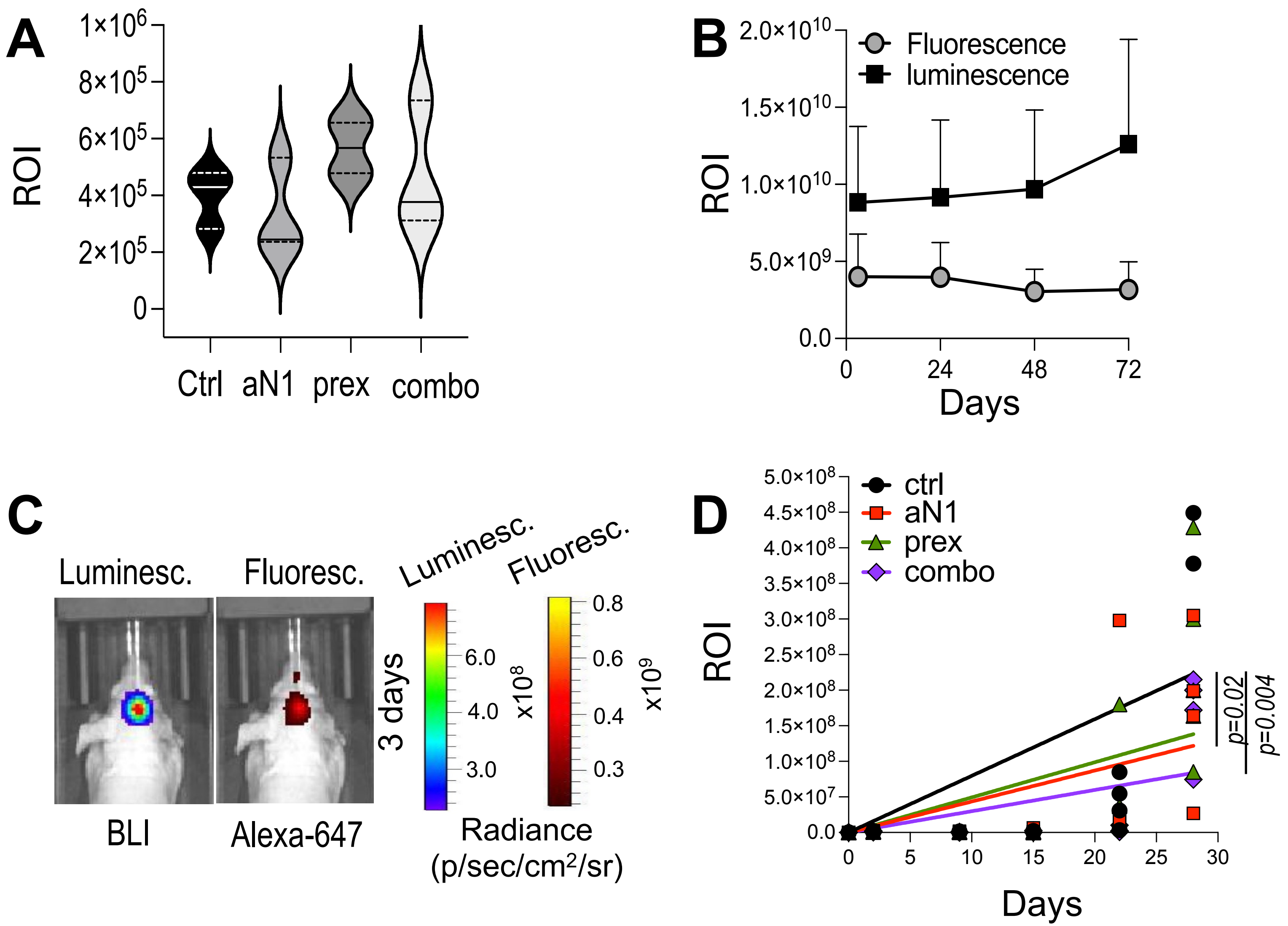

Suppl. Fig. 7: A) Mean radiance intensity of MBMs at day 1 post inoculation as pseudo measure of mean volume in each group of treatment. **B)** anti-N1 crosses the BBB. Radiance intensity of BLI and red fluorescence (anti-N1-alexa 647 conjugated antibody), in 3 extra control brain tumors at day 22 post inoculation. Anti-N1-Alexa 647 conjugated antibody was inoculated IP and radiance followed over 3 days. **C)** Representative pictures of BLI and red fluorescence at 3 days post antibody delivery. **D)** Mean radiance intensity of MBMs over time for each group of treatment. Tumor growth was estimated by a linear regression using mean ROIs as pseudo-tumor volumes. Significant difference in slope coefficient was observed in ctrl vs anti-N1 and prex and ctrl vs combo.
